# Psoas Muscle Infiltration Masquerading Distant Adenocarcinoma

**DOI:** 10.1155/2014/986453

**Published:** 2014-09-22

**Authors:** Kamel A. Gharaibeh, Arnaldo Lopez-Ruiz, Tauqeer Yousuf

**Affiliations:** ^1^Department of Internal Medicine, University of Mississippi Medical Center, 2500 North State Street, Jackson, MS 39216, USA; ^2^Division of Hospital Medicine, Department of Medicine, University of Mississippi Medical Center, 2500 North State Street, Jackson, MS 39216, USA

## Abstract

Malignant metastasis to the psoas muscle is rare. We report a case that clinically mimicked psoas abscess that was subsequently proven to be from metastatic disease secondary to adenocarcinoma of the duodenum. A 62-year-old male presented with a seven-month history of right lower quadrant abdominal pain and progressive dysphagia. CT scan of abdomen-pelvis revealed a right psoas infiltration not amenable to surgical drainage. Patient was treated with two courses of oral antibiotics without improvement. Repeated CT scan showed ill-defined low-density area with inflammatory changes involving the right psoas muscle. Using CT guidance, a fine needle aspiration biopsy of the right psoas was performed that reported metastatic undifferentiated adenocarcinoma. Patient underwent upper endoscopy, which showed a duodenal mass that was biopsied which also reported poorly differentiated adenocarcinoma. In this case, unresponsiveness to medical therapy or lack of improvement in imaging studies warrants consideration of differential diagnosis such as malignancy. Iliopsoas metastases have shown to mimic psoas abscess on their clinical presentation and in imaging studies. To facilitate early diagnosis and improve prognosis, patients who embody strong risk factors and symptoms compatible with underlying malignancies who present with psoas imaging concerning for abscess should have further investigations.

## 1. Introduction

The most frequent tumor of the duodenum is adenocarcinoma [1, 2]. Adenocarcinoma of the duodenum may arise from duodenal polyps observed in familial polyposis or Gardener's syndrome or be associated with celiac disease [3, 4]. The tumor can be located in any part of the duodenum but is most frequently found in the descending part. The most common metastatic sites of duodenal carcinoma are liver, lungs, lymph nodes, and peritoneum. To our knowledge, metastasis to skeletal muscle from duodenal carcinoma has never been described. Furthermore, skeletal muscle is a rare site for metastases which manifest clinically, but has a slightly higher reported autopsy incidence of 16–25% [5, 6]. Leukemia, high grade lymphomas, and carcinoma (originating from cervix, ovary, colon, stomach, lung, breast, and kidney) are the most frequent primary sources [5–7]. Skeletal metastatic lesions usually mimic the clinical presentation of abscess or hematomas [5, 8]. This fact constitutes the most frequent reason for delaying appropriate therapy of the underlying malignant process [5]. The purpose of this report is to describe perhaps the first case in the literature of iliopsoas metastasis from duodenal adenocarcinoma.

## 2. Case Report

**Figure 1:**
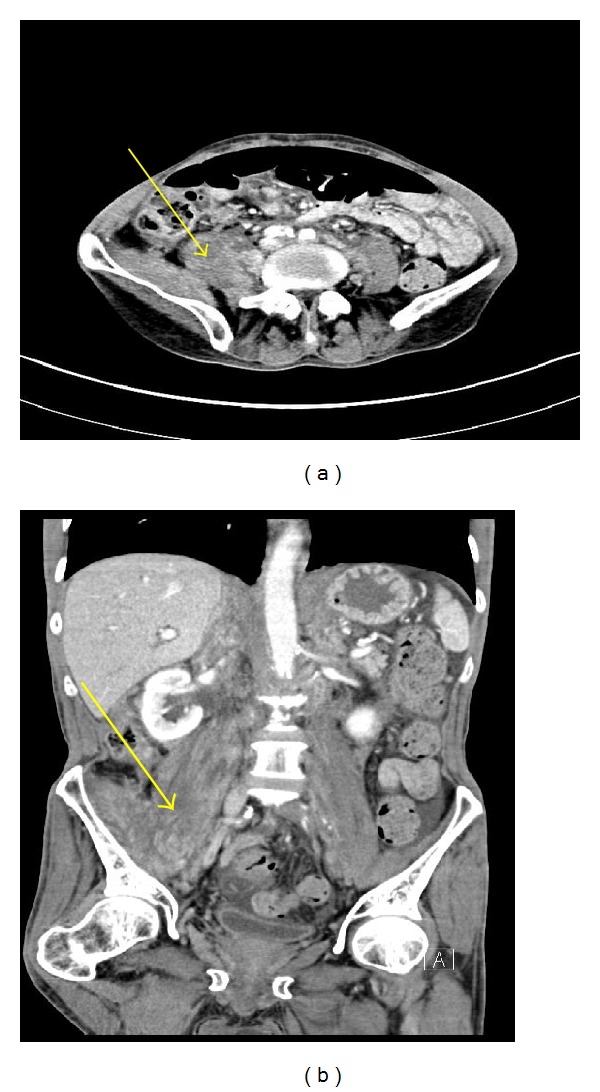
(a) Contrast-enhanced axial CT image through upper pelvis demonstrate enlarged, heterogeneously enhancing right psoas and iliacus muscles with the muscles demonstrating increased and prominent striations (as pointed by arrow). Left psoas is normal. (b) Contrast-enhanced coronal CT image through the abdomen and the pelvis demonstrate enlarged, heterogeneously enhancing right psoas and iliacus muscles (as pointed by arrow) with lateral and anterior displacement of right kidney. No fluid collection is seen.

**Figure 2:**
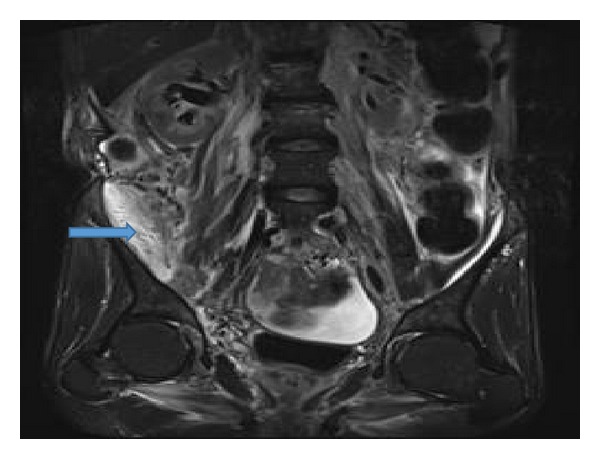
T2 coronal MR imaging showed enlarged predominantly hyperintense right psoas and iliopsoas (as pointed by arrow) without any discrete fluid collection. Right kidney displaced anteriorly and laterally.

**Figure 3:**
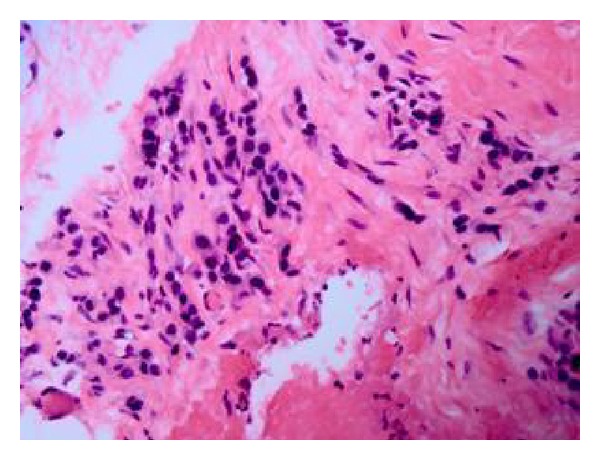
(H&E, 20x) CT-guided psoas muscle biopsy showing tumor cells in the dense fibroconnective tissue with background lymphocytes. The individual tumor cells are atypical, pleomorphic, and hyperchromatic in an infiltrative pattern and were cytokeratin positive, confirming the diagnosis of metastatic poorly differentiated carcinoma.

**Figure 4:**
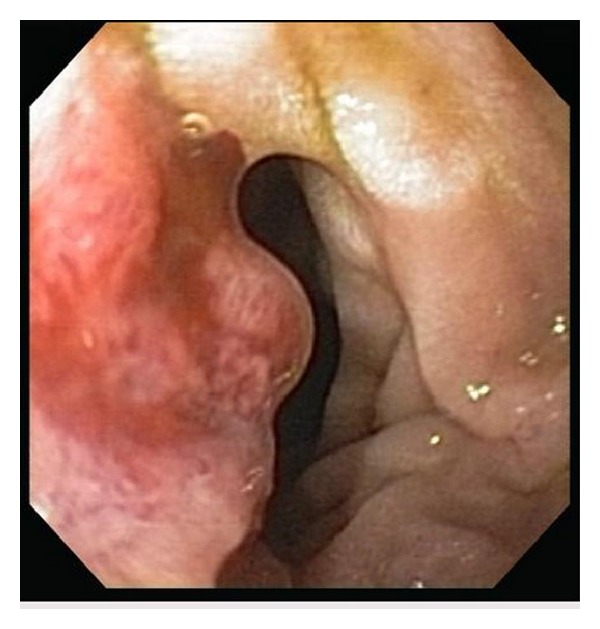
Duodenal mass.

**Figure 5:**
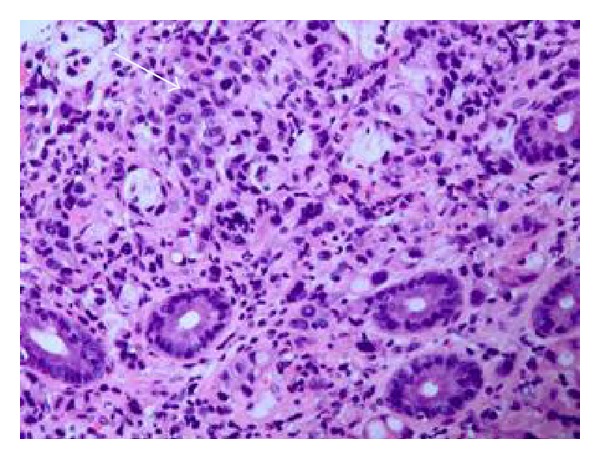
(H&E, 20x) Duodenal mass biopsy showing tumor cells in an infiltrative pattern interspersed within intestinal mucosa. The cells are hyperchromatic and pleomorphic and show abortive gland formation (arrow) consistent with poorly differentiated adenocarcinoma.

A 62-year-old Caucasian male with history of chronic obstructive pulmonary disease, gastroesophageal reflux disease, and knee osteoarthritis presented with seven months history of gradual, dull, and constant abdominal pain affecting the right lower quadrant (RLQ). The pain radiated to his right groin and down his anterior right thigh. Patient also reported 10 months history of mild but persistent epigastric discomfort, dysphagia, and odynophagia with solids and liquids. The patient denied history of fever or chills but did report significant weight loss (>10 lb) and night sweats over the last six weeks. The patient had an extensive tobacco history of two packs-per-day for fifty years, as well as an extensive alcohol history despite abstinence the preceding months. Three months prior to admission to our service, patient underwent laparoscopic cholecystectomy due to the abdominal pain that was described above. However, his abdominal pain persisted postoperatively. Patient presented to our emergency department and was sent home on oral antibiotics for iliopsoas phlegmon as per CT findings thought to be secondary to infection. He presented for the second time two weeks later complaining of severe abdominal pain; repeat CT scan of abdomen and pelvis did not show any significant change in the iliopsoas phlegmon. Vital signs were within normal. Physical exam showed a cachectic man that appeared older than stated age. Abdomen was soft, nondistended and with moderate tenderness to palpation in RLQ and suprapubic area with no evidence of peritoneal inflammation. There were normal bowel sounds and no hepatosplenomegaly. The rest of the physical exam was normal. Initial laboratory results on admission revealed hemoglobin to be 12.6 g/L and otherwise normal CBC. Renal function was within normal. Total protein was 5.8 g/L, albumin was 2.9 g/L, alkaline phosphatase was 1619 mg/dL, ALT was 51 UI, AST was 43 UI, GGT was >1200 u/L, C-reactive protein was 3.7 mg/dL (normal range <5 mg/dL), and ESR was 52. Blood cultures were negative. An abdominal ultrasound reported normal liver measuring 15 cm, no definitive intrahepatic biliary dilatation and mild extrahepatic biliary dilation (CBD measuring 10 mm) that was likely related to cholecystectomy, suggested as not significant. Ultrasound was not done to evaluate psoas muscle. A repeated CT scan of abdomen-pelvis reported ill-defined areas of low density involving the right psoas muscle from origin to insertion and inflammatory changes along the right psoas muscle and right iliacus muscle (Figures 1(a) and 1(b)). Soft tissue lumbar MRI showed persistent thickening and heterogeneity of the entirety of the right iliopsoas musculature. Axial T1 post contrast MRI (images of the lumbar spine) showed normal appearing muscle segments on the left side. Significantly enlarged and enhancing right psoas and paraspinal musculature on the right side (Figure 2). The differential diagnoses suggested by the radiologist were infectious process versus neoplastic infiltration. Given the malignant suspicious of the lesion, using CT guidance, a fine needle aspiration biopsy of the right psoas mass was performed. The core biopsy sections revealed atypical cells with high nuclear cytoplasmic ratio and hyperchromasia in an infiltrative pattern and were positive for cytokeratin immunostain confirming the diagnosis of metastatic poorly differentiated carcinoma (Figure 3). CT of head, neck, and chest was done because of significant smoking history, however, it was negative for any abnormality suggestive of malignancy. Given progressive dysphagia, patient underwent upper endoscopy, which revealed esophageal stricture that was dilated. Stomach showed no endoscopic evidence of ulcers or masses. Upon reaching the duodenum, ERCP was attempted for elevated alkaline phosphatase and GGT using regular scope but cannulation was not achieved; near the ampulla, a protruding luminal mass was seen in the second portion of the duodenum (Figure 4). Duodenal biopsy was taken which showed similar tumor cells in an infiltrative pattern consistent with poorly differentiated adenocarcinoma (Figure 5). Upon discussing the results with the patient, he refused any type of therapy and requested palliative care. He was discharged to hospice care facility.

## 3. Discussion

The clinical presentation of this patient was compatible with a “malignant psoas syndrome” that is usually secondary to infiltrating and metastatic processes [9]. Two very common differential diagnoses that must be considered in this condition are psoas abscess or psoas hematoma. However, in situations when psoas abscess is considered, the poor response to antibiotics and lack of improvement in the imaging studies after 3-4 weeks of therapy should encourage further investigation to rule out a malignant process [10]. It has been reported that 40% of the lesions initially considered as psoas abscess were found later to be lymphomas, sarcomas, or metastasis [8]. Our patient did not present any risk factor usually associated with psoas abscess [10, 11]. The clinical presentation with no fever, absence of leukocytosis, reduced C-reactive protein, negative blood cultures, and null improvement with antibiotics supported a noninfectious process. Given the strong history of smoking and chronic gastrointestinal symptoms (dysphagia and odynophagia) associated with significant weight loss, we ordered a CT scan abdomen-pelvis which showed an extensive enhancing lesion in the lower area of the right psoas. This study was not confirmatory for an abscess and was unable to show any endoluminal lesion in the small bowel or a direct extension from a tumoral process. It has been reported that muscle metastases appear as focal or diffuse areas of muscle enlargement, occasionally isodense, in noncontrast CT [12]. Contrast enhancement may provide further information on disease extent, but is nonspecific for the diagnosis of malignancy, as more than 50% of inflammatory diseases show rim enhancement postcontrast administration [13]. Some reports have shown that CT imaging give poor accuracy and sensitivity in the differentiation of iliopsoas neoplasm from abscess or hemorrhage [14]. We then evaluated this lesion by MRI, which highly suggested a malignant infiltration. Use of MRI for the diagnosis of psoas muscle disorders has been previously reported to present more than 90% sensitivity and to present higher specificity than CT for detection of sarcomas and metastatic lesions [15, 16]. In order to confirm our suspicions, we performed a CT guided biopsy which showed a metastatic poorly differentiated adenocarcinoma. Metastasis of carcinoma to the skeletal muscle is uncommon and generally found in patients with advanced-stage neoplasm. The prevalence of skeletal metastasis in the population with cancer is extremely low (0.03%) [17]. In addition, 90% of patients with skeletal muscle metastasis were found to have widespread metastatic lesions, brain, bone, and lungs being the most commons sites [18]. It is thought that constant muscle movement and the muscle's capability to remove lactic acid and free oxygen radicals produced by tumor contribute to the resistance of skeletal muscles for the metastatic process [19, 20]. Given the clinical presentation of gastrointestinal symptoms (dysphagia) and negative CT chest for lung malignancy, GI workup was started with an upper endoscopy, which was performed to locate the primary lesion. This study showed a large endoluminal mass in the second portion of the duodenum that was biopsied and later reported as poorly differentiated adenocarcinoma. To our surprise, this duodenal mass was not seen in the upper endoscopy performed in outside facility. This type of tumor is an extremely uncommon malignant lesion. The peak of frequency is the sixth decade. Signs and symptoms are unspecific and include abdominal pain (60%), weight loss (70%), nausea and vomiting (30%), jaundice (30%), and hemorrhage (40%) [21]. A palpable abdominal mass is found in less than 5% of the patients [22]. Five to 40% of the patients have distant metastases or peritoneal seeding at the time of diagnosis [23]. Complete surgical resection is the only hope for cure. Pancreatoduodenectomy and various types of lymphadenectomies have been advocated as the surgical procedure of choice because they offer the possibility of regional lymph node resection [24, 25]. Radiotherapy and chemotherapy have been used in few cases most often as an adjuvant postoperative treatment with no improvement in outcome. The 5-year survival rate varies widely according to the series published, but is generally reported to be >40% in case of curative resection and to less than 5% in cases of metastatic disease [23–25].

In summary, iliopsoas metastases may mimic psoas abscess or hematomas, both clinically (lower abdominal pain, decreased hip excursion) as well as at imaging (asymmetric swelling of psoas muscles with or without low attenuation regions showing heterogeneous enhancement that can be either unilateral or bilateral). In a patient who presents clinically with hip pain, flexion deformity, and limited extension, the characteristic imaging findings and relevant history will usually lead to the correct diagnosis. However, in a patient with unknown primary tumor but with risk factors (smoking) and chronic symptoms compatible with underlying malignancy who shows abnormalities on psoas muscle imaging, the possibility of metastases, although uncommon, should be considered.
